# Vitrectomy with Air Endotamponade for Traumatic Cyclodialysis

**DOI:** 10.1155/2020/3742306

**Published:** 2020-09-09

**Authors:** Anan Wang, Zhenquan Zhao

**Affiliations:** ^1^Department of Ophthalmology, Affiliated Eye Hospital of Nanchang University, Nanchang, China; ^2^Department of Ophthalmology, Eye Hospital of Wenzhou Medical University, Wenzhou, China

## Abstract

**Background:**

Various techniques have been described for repairing cyclodialysis clefts, but there is no consensus regarding the optimal treatment. This study investigated the clinical efficacy of a novel surgical approach that is used to manage traumatic cyclodialysis.

**Methods:**

We prospectively enrolled 7 patients (7 eyes) with traumatic cyclodialysis of the concomitant lens and other vitreous diseases. Ultrasound biomicroscopy was used to diagnose cyclodialysis, and all eyes underwent pars plana vitrectomy with air endotamponade. The main outcome measures were postoperative anatomical success rates, best-corrected visual acuity (BCVA), and intraocular pressure (IOP).

**Results:**

All patients were male, and their age ranged from 46 to 64 years (mean: 54.3 years). After the surgical intervention, the extent of the cyclodialysis cleft ranged from 1 to 4 clock hours (mean: 2.3 clock hours) and the detached ciliary body of all cases was completely restored; the anatomical success rate was 100%. The BCVA significantly increased from 1.40 ± 0.49 to 0.42 ± 0.31 (*P* = 0.002). The IOP increased from 8.91 ± 1.77 to 14.67 ± 6.38, but the difference was not significant (*P* = 0.056). The postoperative IOP of most patients was temporarily elevated or lowered after surgery. At the last follow-up, there were still two abnormal cases, including one with ocular hypertension and one with hypotony.

**Conclusions:**

This study revealed that vitrectomy with air endotamponade is an effective and minimally invasive alternative surgical approach for small traumatic cyclodialysis clefts.

## 1. Introduction

Cyclodialysis results from the separation of the longitudinal ciliary muscle fibres from the scleral spur, which leads to hypotony, a decrease in the production of aqueous fluids, and the opening of an additional drainage pathway of the aqueous humour into the suprachoroidal space. Secondary complications include refractory changes, the development of cataracts, optic disk swelling, choroidal detachment, macular oedema, and vision loss [1, 2]. Cyclodialysis can be caused by ocular trauma or surgical injury, and gonioscopy and ultrasound biomicroscopy (UBM) are used for its diagnosis. Furthermore, numerous therapeutic strategies have been reported for the treatment of cyclodialysis; the underlying mechanisms of these treatments involve the reattachment of the ciliary body to the sclera and restoration of the intraocular pressure (IOP) [3]. Currently, at least twenty different techniques, including systemic steroids, topical steroids, atropine medical treatments, and a variety of surgical treatments, such as injection of viscoelastic materials or blood plasma, laser photocoagulation, diathermy, cyclocryotherapy, cyclopexy, sclera-sutured capsular tension rings, intraocular lenses, scleral buckling, and vitrectomy with endotamponade, have been proposed [1, 2]. Direct cyclopexy has been considered the most effective treatment for cyclodialysis [1]. However, treatment should be decided on a case-to-case basis, according to the extent of the cleft and the presence of concomitant ocular diseases. Noninvasive medical treatments are recommended in cases of small and medium clefts. Cataracts and vitreoretinal surgery with endotamponade should be combined for large cyclodialysis clefts or in cases with a concomitant pathology [1].

The most common cause of traumatic cyclodialysis is severe contusion from blunt ocular trauma [3]. Other ocular comorbidities, including the development of cataracts, lens dislocation, vitreous haemorrhaging, optic disk swelling, macular oedema, and retinal and choroidal detachment, may be present and require medical attention. Due to the effects of media opacity, UBM is more widely performed for the diagnosis of traumatic cyclodialysis compared to gonioscopy. Furthermore, vitrectomy with endotamponade is recommended for the treatment of traumatic cyclodialysis that presents with a concomitant posterior segment pathology [1]. However, silicone oil or expansile gas are the most widely used vitreous tamponade materials [3–8], and their use is often combined with additional procedures, such as cryotherapy [4, 5, 7, 9], transscleral diathermy [6], capsular tension ring placement [7], and cyclopexy [6, 10]. According to previous reports, silicone oil and expansile gas may lead to complications, such as the development of cataracts, ocular hypertension, and emulsification. Compared to air, they are expensive, require a longer tamponade duration, and fixed head position, and are associated with more complications [11, 12]. Also, performing additional surgical procedures induces more discomfort, longer intraoperative times, and more surgical damage. However, there are no known studies that have used vitrectomy with air endotamponade to treat cyclodialysis.

Therefore, the purpose of this study was to investigate the efficacy of vitrectomy with air endotamponade, without additional procedures, for the treatment of traumatic cyclodialysis.

## 2. Materials/Subjects and Methods

### 2.1. Patients and Ethical Considerations

We prospectively enrolled consecutive 26 patients suffering from traumatic cyclodialysis who were treated at the Eye Hospital of Wenzhou Medical University or Affiliated Eye Hospital of Nanchang University between November 2018 and May 2020. We included patients who had cyclodialysis with lens dislocation, vitreous haemorrhaging, retinal contusion, or choroidal detachment following ocular trauma. We excluded patients who were diagnosed with severe choroidal or retinal diseases (17 cases), or lost to follow-up (2 cases). The study was approved by the Ethics Committee of the Affiliated Eye Hospital of Nanchang University and was conducted in accordance with the tenets of the Declaration of Helsinki. All patients were informed about the procedures of the surgery and possible merits and risks before providing written consent to participate in the study.

### 2.2. Patient Examinations

Each patient underwent a complete ocular examination before vitrectomy, which was performed by a surgeon with 15 years of experience at both hospitals. This examination included the best-corrected visual acuity (BCVA), IOP measurements (pneumotonometer TX-20, Canon Inc., Tokyo, Japan), slit-lamp photography, gonioscopy, B-scan ultrasound, and UBM (HF 35-50, OTI, Toronto, Canada). BCVA was determined using the standard logarithmic vision chart, and the value was transferred to the digital logarithm of the minimum angle of resolution (LogMAR) scale for statistical analysis; the visual acuity values of counting fingers, hand movements, light perception (LP), and no LP were substituted by logMAR values of 1.7, 2.0, 2.5, and 3.0, respectively [13, 14]. IOP was recorded as the average of three measurements. B-scan ultrasound was used to evaluate the condition of the posterior segment, and patients with retinal detachment were excluded from the study. UBM was used to assess the size of the cyclodialysis cleft and was performed at least three days later after debridement surgery in open-globe injuries.

### 2.3. Treatments and Postoperative Course

All patients were prescribed topical steroids and antibiotics after injury. Topical atropine 1% was administered immediately after the diagnosis of cyclodialysis. To remove the vitreous and haemorrhage, all patients underwent a 3-port 23-gauge transconjunctival pars plana vitrectomy. Moreover, a pars plana lensectomy, phacofragmentation, or phacoemulsification were performed in cases with lens dislocation or developing cataracts. Repair for iris laceration and endolaser photocoagulation for retinal breaks was performed when necessary. Air-fluid exchange was accomplished in all patients.

The patients were asked to maintain a fixed head position for one week postoperatively to hold the cleft; during this time, topical corticosteroids and antibiotics were prescribed. Postoperative examinations were planned for days 1, 2, and 3, week 2, and months 1, 3, 6, and 12. Twelve months after surgery, follow-ups were scheduled annually; during these examinations, clinical follow-up data, including anatomical outcomes, BCVA, IOP, IOP spike (IOP ≥25 mmHg) [15], and hypotony (IOP≤5 mmHg), were recorded [1]. Gonioscopy and UBM were performed at least two weeks later postoperatively. All patients were followed-up for at least three months; the mean follow-up period was 3.90 ± 1.15 months (range: 3 to 6 months).

### 2.4. Statistical Analyses

All statistical analyses were performed using SPSS software v19.0 (IBM; Armonk, New York, United States). All continuous variables were tested for normality using the Kolmogorov–Smirnov test and are expressed as mean ± standard deviation. The paired *t*-test was used to compare preoperative and postoperative clinical results. *P* values <0.05 were considered statistically significant.

## 3. Results

We analysed 7 eyes (3 right eyes) of 7 patients (7 males) with a mean age of 54.3 years (range: 46–64 years). The causes of injury included car accident (1/7, 14.3%), stone (1/7, 14.3%), wood (1/7, 14.3%), fireworks explosion (1/7, 14.3%), and iron (3/7, 42.9%); injury occurred due to contusion in six cases and penetration in one case. All patients had additional ocular trauma complications, including iris injury (5/7, 71.4%), lens dislocation (5/7, 71.4%), traumatic cataracts (4/7, 57.1%), vitreous haemorrhaging (6/7, 85.7%), choroidal detachment (2/7, 28.6%), retinal contusion (2/7, 28.6%), and retinal hole (1/7, 14.3%). Preoperative and postoperative ocular findings are presented in Table 1.

The average length of time between injury and vitrectomy surgery was 12.9 days (range: 5–31 days). The mean extent of cyclodialysis was 2.3 clock hours (range: 1 to 4 clock hours). The mean preoperative BCVA was 1.40 ± 0.49 (range: 2 to 0.70). The mean preoperative IOP was 8.91 ± 1.77 mmHg (range: 5.2 to 10.5 mmHg).

In this study, iridodialysis repair was performed in 2 out of 7 cases. Lens extraction, including lensectomy (3/7, 42.9%) and phacofragmentation (4/7, 57.1%), were performed in all eyes. Retinal lasers were applied for a retinal hole in one patient. Air during vitreous tamponade (after vitrectomy) and air-fluid exchange were utilized in all patients.

**Figure 1:**
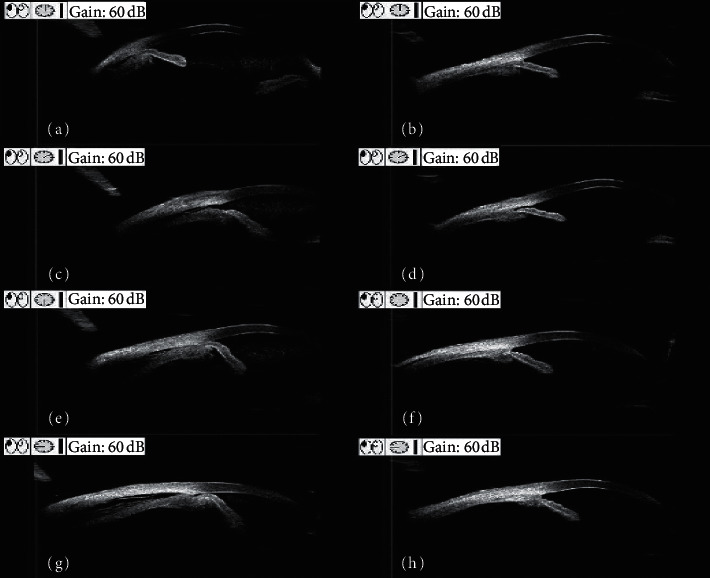
Preoperative and postoperative ultrasound biomicroscopy (UBM) images of Patient 1. (a, c, e, g) Preoperative UBM findings show the cyclodialysis clefts at the 2 o'clock position; the other ciliary body was detached. (b, d, f, h) Postoperative UBM findings show that the cyclodialysis cleft is closed two weeks after surgery.

After vitrectomy and air endotamponade, all cyclodialysis was recovered; the anatomical success rate was 7/7 (100%).  Figure 1 shows a representative case (Patient 1). The postoperative BCVA significantly increased; the mean value was 0.42 ± 0.31 (range: 0.8 to 0.1; *P* = 0.002). The postoperative IOP also increased; the mean value was 14.7 ± 6.38 mmHg and ranged from 4.4 to 24.9 mmHg, but there were no significant differences compared to the preoperative IOP (*P* = 0.056). Comparisons of the preoperative and postoperative BCVA and IOP values are presented in Table 2.

**Figure 2:**
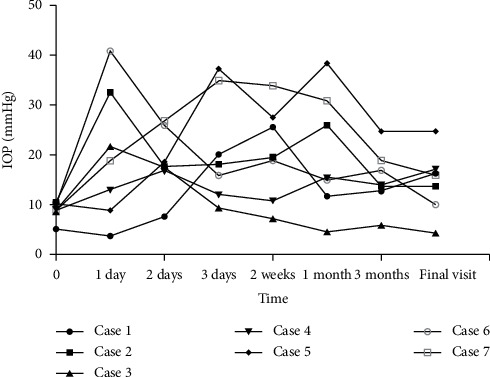
Postoperative intraocular pressure (IOP) profile following vitrectomy with air endotamponade in all cases.

The postoperative IOP was temporarily elevated or lowered after surgery in most cases; the IOP values recorded during follow-up are shown in Figure 2. Antihypertensive agents were used for an elevated IOP, while topical corticosteroids were used for hypotony. However, at the last follow-up, there were still two abnormal cases, including one with ocular hypertension (Patient 5) and one with hypotony (Patient 3). Additionally, two patients had peripheral anterior synechiae (PAS), Patient 5 had a 360°-PAS, and Patient 7 had a 90°-PAS.

## 4. Discussion

This study evaluated the clinical efficacy of vitrectomy with air endotamponade for the treatment of traumatic cyclodialysis. The results revealed that vitrectomy with air endotamponade is an effective and minimally invasive surgical approach for treating small traumatic cyclodialysis clefts presenting with lens and vitreous diseases.

Cyclodialysis is a very rare disease, making it difficult to conduct large studies; most studies have presented only a few cases or single case reports, with the rates ranging between 1 and 11% [16]. In our study, we prospectively analysed seven patients with small traumatic cyclodialysis clefts. All patients were males; this was likely because males are more susceptible to traumatic injury compared to females [1]. Moreover, cyclodialysis is caused by blunt trauma in most cases. In Agrawal's series, 16 of 17 cases were caused by blunt trauma [17]. Similarly, in our study, 6 of 7 cases were caused by blunt trauma, while 1 case was caused by penetrating trauma.

The main clinical manifestation of cyclodialysis is usually hypotony, and secondary changes to all other ocular tissues are generally attributed to persistent hypotony [1]. However, no cases presented with hypotony in our study, and the mean preoperative IOP was 8.91 ± 1.77 mmHg (range: 5.2 to 10.5 mmHg). These results may be due to additional ocular damage, including inflammation, intraocular haemorrhaging, angle damage, and lens-related injury, which may lead to the presence of white or red blood cells in the anterior chamber or direct injury to the trabecular meshwork or synechial angle-closure [18]. Additionally, despite the additional drainage pathway of the aqueous humour, these changes could hinder the aqueous outflow pathways and lead to no presentation of hypotony.

Some atypical clinical manifestations present with normal IOP make cyclodialysis clefts difficult to diagnose. Therefore, we propose that all traumatic eyes should undergo examinations of the ciliary body to avoid misdiagnosis of cyclodialysis; gonioscopy, UBM, or anterior segment optical coherence tomography should be used for these examinations. However, gonioscopy and anterior segment optical coherence tomography may be affected by anterior segment media opacity. In cases where direct visualization is difficult, high-frequency UBM can be used [17].

Treatment options for cyclodialysis vary from case to case, but the ciliary body can be reattached to the sclera and ocular function can be restored with proper therapy in most cases. In cases of traumatic cyclodialysis, which always presents with other vitreous comorbidities, vitrectomy and endotamponade therapy are the most commonly used treatment regimens. One or more additional procedures are usually performed concurrently to enhance adhesion. In 2013, Dutra Medeiros et al. first reported vitrectomy using silicone oil or expanding gas for endotamponade [3]. In this technique, the endotamponade is used to approximate the detached ciliary body to the sclera and allow scar formation. However, there are no known studies utilizing vitrectomy with air tamponade.

Many ophthalmologists have suggested that noninvasive treatments are effective for small clefts, which are generally less than 3 clock hours [19, 20]. The mean extent of the preoperative cyclodialysis in our series was 2.3 clock hours (range: 1–4 clock hours). Because all patients in our series had additional ocular anterior and posterior segment damage, we chose vitrectomy with air endotamponade to treat cyclodialysis. Postoperatively, the ciliary body was reattached in all cases, and the values for BCVA and IOP were favourable. These results demonstrated that vitrectomy with air endotamponade was efficient for repairing small cyclodialysis clefts. The principle of this treatment method is to apply the property of surface tension and buoyancy of air endotamponade to reattach the cyclodialysis. In a fixed head position, the gas bubble can obstruct the flow of the aqueous humour and hold the detached ciliary body to the sclera, which results in reattachment [9].

Hoerauf et al. first applied air as an intraocular tamponade to treat traumatic cylcodialysis in 1999 [9], but additional cryotherapy was combined to induce fibrin exudation and enhance adhesion. Their mechanism was similar to the principle for treating retinal detachment. Hence, many authors believe that inducing fibrin exudation is necessary. However, topical atropine therapy can also be used to close the cyclodialysis; similar to the role of the gas bubble, it can locate the ciliary body to the sclera and enhance reattachment [19]. Furthermore, spontaneous closure of the cleft may reveal the tendency of the tissue to self-heal [21]. Therefore, based on our results, we conclude that vitrectomy with air endotamponade is a sufficient treatment method for small clefts and that additional surgical procedures are not necessary. For extensive clefts, a longer endotamponade or more invasive procedures may be necessary.

When treating cyclodialysis, it is important to recover the anatomical integrity of the eye, reestablish visual functions, and maintain a normal IOP. However, successful cleft reattachment often does not correspond with a normal IOP, especially in patients with traumatic cyclodialysis. IOP is associated with aqueous humour circulation; traumatic changes in the function of the ciliary body, the presence of direct injury of the trabecular meshwork, angle recession, or PAS will induce abnormalities of the aqueous humour dynamics, which will affect IOP [17, 18]. Ocular hypertension and hypotony are also common after vitrectomy surgery. In our study, although all cases presented a diverse IOP profile during the early postoperative stage, most of them were within the normal range. One patient was hypertensive (Patient 5) and one was hypotonic (Patient 3) at the last follow-up. Ocular hypertension after ocular trauma and vitrectomy is common. It likely occurs due to a shallower anterior chamber [22], PAS [22], or trabecular meshwork damage [10, 18]. In our study, ocular hypertension likely occurred in Patient 5 due to 360°-PAS. Hypotony might occur due to dysfunction of the ciliary body [8] or aphakia [23]; the hypotony observed in Patient 3 in the present study is thought to have occurred due to a combination of these mechanisms. Hence, the IOP and closure of the cleft cannot be the only criteria for success. Therefore, we believe an abnormal IOP or cleft does not need to be repaired again if morphological and functional complications are not present.

A potential concern with our method of repair cyclodialysis is the PAS. In our study, two patients suffered PAS after undergoing vitrectomy with air endotamponade. Although traumatic damage may lead to PAS [24], we tend to believe it is a possible complication of air endotamponade. The two cases with PAS had 360° pupillary sphincter damage and 120° iris injury, respectively; after ocular trauma, these conditions caused a dilated pupil and peripheral iris accumulation. The gas bubble formed after vitrectomy with air endotamponade can hold the iris to the cornea, promote the formation of anterior synechiae, and lead to secondary angle-closure glaucoma [25]. To avoid this situation, we decided that viscoelastic is a good choice. During the vitrectomy, viscoelastic can be injected to the anterior chamber to avoid postoperative iridocorneal synechiae, especially in patients with iris injury.

This study has a few limitations that should be addressed. First, because traumatic cyclodialysis with mild vitreous or retinal diseases is extremely rare, the sample was small. Furthermore, the follow-up period was short, and a control group was not included for comparison. Despite these limitations, this is the first known study to report the use of vitrectomy with air tamponade for the treatment of traumatic cyclodialysis. We plan to conduct additional studies with larger sample sizes, bigger cyclodialysis clefts, longer follow-up periods, and appropriate control groups to better evaluate the efficacy of this approach.

In conclusion, our study revealed that vitrectomy with air endotamponade is an effective and minimally invasive alternative approach to treat small traumatic cyclodialysis clefts with concomitant lens and vitreous diseases. Furthermore, it is not necessary to combine additional procedures, such as cryotherapy, endophotocoagulation, transscleral diathermy, or cyclopexy. The results of this study may help surgeons select the appropriate treatment method for the closure of cyclodialysis.

## Figures and Tables

**Table 1:** Preoperative and postoperative features of patients.

Case no.	Age/sex/eye	Injury cause	Injury type	Time from injury to surgery (days)	Length of follow-up (months)	Extent of cyclodialysis cleft (clock-hours)	Concomitant ocular traumatic diseases	Procedure	Best-corrected visual acuity (LogMAR)		IOP (mmHg)		Anatomical success	IOP spike	Hypotony
									Preoperative	Postoperative	Preoperative	Postoperative			
1	56/M/R	Car accident	Contusion	6	4.3	1	LD, VH, CD	PPV, lensectomy, air	0.70	0.10	5.2	16.4	Yes	Yes	Yes
2	46/M/L	Stone	Contusion	22	3	4	Iridodialysis, LD, TC, VH,	PPV, IR, lensectomy, air	1.00	0.22	10.5	13.8	Yes	Yes	No
3	56/M/L	Iron nail	Penetrating	8	6	2	IL, LD, TC, VH, CD	PPV, phacofragmentation, air	2.00	0.30	8.7	4.4	Yes	No	Yes
4	52/M/L	Iron block	Contusion	7	4.7	3	Iridodialysis, LD, TC, VH, RH, RC	PPV, IR, phacofragmentation, RL, air	1.70	0.10	9.2	17.2	Yes	No	No
5	55/M/L	Iron block	Contusion	5	3.3	2	Iridodialysis, LD, TC, VH, RC	PPV, phacofragmentation, air	1.70	0.80	10.3	24.9	Yes	Yes	No
6	51/M/R	Wood	Contusion	31	3	1	CA, LD, TON	PPV, lensectomy, air	1.00	0.70	9.5	10.0	Yes	Yes	No
7	64/M/R	Fireworks explosion	Contusion	11	3	3	Iridodialysis, LD, VH	PPV, phacofragmentation, air	1.70	0.70	9.0	16.0	Yes	Yes	No

M, male; R, right; L, left; CA, corneal abrasion; CD, choroidal detachment; IL, iris laceration; LD, lens dislocation; RC, retinal contusion; RH, retinal hole; TC, traumatic cataract; TON, traumatic optic neuropathy; VH, vitreous haemorrhage; IR, iridodialysis repair; PPV, pars plana vitrectomy; RL, retinal laser; IOP, intraocular pressure.

**Table 2:** Comparison of preoperative and postoperative values.

	Preoperative	Postoperative	*P* value
BCVA (LogMAR)	1.40 ± 0.49	0.42 ± 0.31	0.002^*∗*^
IOP (mmHg)	8.91 ± 1.77	14.67 ± 6.38	0.056

Continuous variables are expressed as mean ± standard deviation. BCVA: best-corrected visual acuity; IOP: intraocular pressure; ^*∗*^*P* < 0.05.

## Data Availability

The datasets generated and analysed during the present study are available from the corresponding author upon reasonable request.
